# Sleep deprivation induces corneal endothelial dysfunction by downregulating Bmal1

**DOI:** 10.1186/s12886-024-03524-4

**Published:** 2024-06-21

**Authors:** Yani Wang, Qun Wang, Shengqian Dou, Qingjun Zhou, Lixin Xie

**Affiliations:** 1 Eye Institute of Shandong First Medical University, Qingdao Eye Hospital of Shandong First Medical University, 5 Yan er dao Road, Qingdao, 266071 China; 2 State Key Laboratory Cultivation Base, Shandong Provincial Key Laboratory of Ophthalmology, Shandong First Medical University, Shandong, China; 3 School of ophthalmology, Shandong First Medical University, Shandong, China

**Keywords:** Corneal endothelium, Sleep deprivation, Bmal1, Mitochondrial function

## Abstract

**Background:**

Sleep deprivation (SD) is a common public health problem that contributes to various physiological disorders and increases the risk of ocular diseases. However, whether sleep loss can damage corneal endothelial function remains unclear. This study aimed to determine the effect and possible mechanism of SD on the corneal endothelium.

**Methods:**

Male C57BL/6J mice were subjected to establish SD models. After 10 days, quantitative RT-PCR (qRT-PCR) and western blot or immunostaining for the expression levels of zonula occludens-1 (ZO-1), ATPase Na+/K + transporting subunit alpha 1 (Atp1a1), and core clock genes in the corneal endothelium were evaluated. Reactive oxygen species staining and mitochondrial abundance characterized the mitochondrial function. The regulatory role of Bmal1 was confirmed by specifically knocking down or overexpressing basic helix-loop-helix ARNT like 1 protein (Bmal1) in vivo. In vitro, a mitochondrial stress test was conducted on cultured human corneal endothelial cells upon Bmal1 knockdown.

**Results:**

SD damaged the barrier and pump functions of mouse corneal endothelium, accompanied by mitochondrial dysfunction. Interestingly, SD dramatically downregulated the core clock gene Bmal1 expression level. Bmal1 knockdown disrupted corneal endothelial function, while overexpression of Bmal1 ameliorated the dysfunction induced by SD. Mitochondrial bioenergetic deficiency mediated by Bmal1 was an underlying mechanism for SD induced corneal endothelial dysfunction.

**Conclusion:**

The downregulation of Bmal1 expression caused by SD led to corneal endothelial dysfunction via impairing mitochondrial bioenergetics. Our findings offered insight into how SD impairs the physiological function of the corneal endothelium and expanded the understanding of sleep loss leading to ocular diseases.

**Supplementary Information:**

The online version contains supplementary material available at 10.1186/s12886-024-03524-4.

## Background

High-quality sleep plays an important and prominent role in human physical health. In modern society, sleep disorders have become a common public health problem worldwide [1]. Surveys of sleep quality showed that up to 35% of adults and nearly 32.53% of high school students have poor-quality sleep [2]. Insufficient sleep can cause various diseases, such as diabetes, obesity, and vascular diseases [3]. In the eye, previous studies have reported that sleep deprivation (SD) is a major risk factor for ocular surface diseases, such as dry eye and corneal epithelial lesions [4–7]. Therefore, the harm of sleep loss on ocular health has drawn the attention of ophthalmologists.

The human corneal endothelium is a layer of single cells located on the posterior corneal surface that plays an important role in the maintenance of corneal transparency [8]. Human corneal endothelial cells have a limited capacity for regeneration and proliferation, and damage to their barrier and pump functions as a result of ageing and diseases can lead to irreversible corneal oedema, resulting in impairment of visual acuity [9, 10]. Therefore, exploring the underlying pathophysiology of corneal endothelial diseases can contribute to a better understanding of the regulation and protection of corneal endothelial function. Emerging evidence has shown that patients with sleep disorders (such as severe sleep apnoea) exhibit abnormal morphology of the corneal endothelium [11], whereas the effects of sleep disorders on the corneal endothelial function remain largely unknown.

Brain and muscle aryl hydrocarbon receptor nuclear translocator‑like protein 1 (Bmal1) and circadian locomotor output cycles kaput (Clock) are the key components of the core molecular clocks [12, 13], and they have been found to exist in peripheral tissues, contributing to maintain various physiological homeostatic processes [14]. The core clock genes are expressed in many ocular tissues, such as retina [15], lens [16], lacrimal gland [17], iris-ciliary body complex [18], corneal epithelium [19], and the corneal endothelium [20]. However, the specific role of core clock genes involved in maintaining ocular health has not been completely clarified. Recent studies have reported that sleep loss can alter the expression of core clock genes, and causes the accumulation of reactive oxygen species (ROS), leading to extra orbital lacrimal gland dysfunction [21], implying the core clock genes are involved in the regulating ocular homeostasis under sleep loss conditions.

In this study, we found that SD disrupted corneal endothelial function and downregulated Bmal1 expression. Bmal1 deficiency contributing to accumulated ROS and mitochondrial dysfunction was the potential mechanism that led to damage of endothelial cells under SD conditions. Our findings provide novel insight into the regulation of corneal endothelial function and expand the knowledge of sleep loss leading to ocular diseases.

## Materials and methods

### Animal models

The Medical Ethics Committee of Shandong Eye Institute approved this study, and all experiments were performed in accordance with the Association for Research in Vision and Ophthalmology (ARVO) Statement and the Animal Research: Reporting of In Vivo Experiments (ARRIVE) Guidelines for the use of animals in ophthalmic and vision research. Two hundred and twenty-five male C57BL/6J mice (12 weeks old, 25–28 g) purchased from SPF Biotechnology Co., Ltd. (Beijing, China) were randomly subjected to establish normal control (Ctrl)(*n* = 40) and SD models (*n* = 55), Bmal1 knockdown (KD) (*n* = 35) or overexpression (OV) (*n* = 35) and negative control (NC) (*n* = 60) models in the same and controlled environment (25℃ ± 1, 50–70% humidity, 12 h light/12 h dark cycle).

SD model was established using a sleep disturbance device (XR-XS108) purchased from Xinruan Information Technology Co., Ltd (Shanghai, China), and the method had been reported in previous studies [22, 23]. In brief, mice were placed in a cylinder (30 × 45 cm) in which an interference rod was continuously rotated at a speed of 15 rpm for 10 days (20 h/day). Mice had free access to water and food.

Recombinant adeno-associated virus (AAV) containing the Bmal1-shRNA, NC-shRNA target sequences, OV-Bmal1, and NC sequences were obtained from Genechem (Shanghai, China). Bmal1-shRNA target sequences were GCATCGATATGATAGATA.

A, and NC shRNA target sequences were CGCTGAGTACTTCGAAATG. The AAV solution of 1 µl with a titer of 5 × 10^12^ vg/mL was injected into the ocular anterior chambers of mice after mice were anesthetized using 0.6% pentobarbital sodium by intraperitoneal injection. Protein was extracted from mouse corneal endothelial tissues after one month to detect transfection efficiency. Corneal endothelial tissues in successful model of mice were collected to conduct the subsequent experiments after mice were humanely euthanized by CO_2_ inhalation followed by cervical dislocation.

### Analyses of central corneal thickness (CCT) and endothelial cell density

Methods for calculating the endothelial cell density and CCT were obtained from the previous studies [24, 25]. CCT was measured using optical coherence tomography (OCT, RT-100, Optovue Inc., Fremont, CA, USA). The endothelial cell density was evaluated by two blinded observers using Premier Endothelial Analytics (Konan Medical, Inc., Hasbrouck Heights, NJ, USA). Approximately 60 cells were selected from a 0.2 mm × 0.24 mm size image for analysis.

### qRT-PCR

Total RNA was extracted from mouse corneal endothelial tissues (6 corneal endothelial tissues as one sample) using the RNAprep Pure Micro Kit (Tiangen, Beijing, China), and then reversely transcribed into cDNA employing a single cell sequence specific amplification kit according to the manufacturer’s protocols (Vazyme, Nanjing, China). Total RNA was extracted from human corneal endothelial cell (HCEC) line B4G12 using Trizol (Life Technologies, Carlsbad, CA, USA), and cDNA was synthesized employing the ReverTra Ace™ qPCR RT Kit (Toyobo, Shanghai, China). Quantitative real-time PCR (qRT-PCR) was conducted on the Sequence Detection System (Applied Biosystems, Foster City, CA, USA) using SYBR qPCR Master Mix (Vazyme, Nanjing, China) with a cycling condition of 95 °C for 30 s followed by 40 two-step cycles (95 °C for 10 s and 60℃ for 30 s). Glyceraldehyde-3-phosphate dehydrogenase (Gapdh) was used as an internal reference gene. The primer sequences are provided in Supplementary Table S1.

### Extraction of DNA and mitochondrial DNA (mtDNA) copy number analyses

Total DNA was extracted from mouse corneal endothelial tissues (10 corneal endothelial tissues as one sample) using the Easy Pure genomic DNA kit (Transgenes, Beijing, China) according to the manufacturer’s protocol. The copy numbers of mtDNA genes, including NADH dehydrogenase subunits 1 and 6 (ND1, ND6), were measured by qRT-PCR using haemoglobin β1 (β-globin) as a reference gene, and presented as a ratio of mtDNA to nuclear DNA. The qRT-PCR conditions were consistent with the procedures mentioned above. The primer sequences are provided in Supplementary Table S1.

### Immunofluorescence staining of whole-mount corneas

Whole-mount mouse corneal tissues were fixed in 4% paraformaldehyde for 10 min and then blocked in 10% donkey serum (Solarbio, Beijing, China) for 2 h. Whole corneal tissues were incubated with primary antibodies overnight at 4℃ and subsequently with fluorescein-conjugated secondary antibodies (the antibodies are supplied in Supplementary Table S2). Whole corneal tissues were washed in phosphate-buffered saline (PBS) three times after incubation with antibodies, and counterstained with 4,6-diamidino-2-phenylindole (DAPI) (Solarbio, Beijing, China). The fluorescence staining of the corneal endothelial cells was observed and imaged using a confocal microscope (LSM880, ZEISS, Jena, Germany).

### Western blot analysis

Protein extracted from mouse corneal endothelial tissues (10 corneal endothelial tissues per sample) or cultured HCEC-B4G12 cells was separated on 12% sodium dodecyl sulphate-polyacrylamide gel electrophoresis gels, and then transferred to polyvinylidene fluoride membranes (Millipore, Billerica, MA, USA). The protein membranes were blocked with 5% bovine serum for 2 h, incubated with primary antibodies (the antibodies are provided in Supplementary Table S2) at 4 °C overnight, and subsequently incubated with horseradish peroxidase-conjugated secondary antibodies for 2 h. Images of the protein bands were observed using an electrochemiluminescence kit (Millipore, Billerica, MA, USA) through enzyme-linked chemiluminescence. The protein expression was quantified by Image Lab (version 3.0).

### Staining of ROS

Corneal frozen sections made from fresh mouse cornea were washed with washing solution, and then incubated with dihydroethidium (DHE probe, 1:200) (HR9069, Baiolaibo, China) for 30 min at 37 °C. The fluorescence intensity was observed and captured using a confocal microscope (LSM880, ZEISS, Jena, Germany) after DAPI staining. To detect ROS in HCEC-B4G12 cells, cultured HCECs in 6-well plates were washed and incubated with a DHE probe (1:1000) (HR8685, Baiolaibo, China) for 30 min at 37℃, and then the fluorescence intensity was observed by Eclipse TE2000-U microscope (Nikon, Tokyo, Japan).

### HCEC-B4G12 cells transfection by lentivirus vector

HCEC-B4G12 with a density of 5 × 10^4^ cells/mm^2^ were cultured in a six-well plate at 37℃ and 5% CO_2_ in an incubator. After 24 h, HCEC-B4G12 cells were infected by lentiviral vectors carrying shRNA targeting knockdown BMAL1 (sh-BMAL1, target sequence: agAACCCAGGTTATCCATATT; ctTCTAGGCACATCGTGTTAT) and normal control viruses (sh-NC, contrast sequence: TTCTCCGAACGTGTCACGT) with a titer of 1 × 10^8^ TU/mL according to the manufacturer’s instructions. After 72 h, HCEC-B4G12 cells were collected, and the transfection efficiency was evaluated at the protein level by western blot.

### Mitochondrial respiration analysis

The methods for detecting mitochondrial metabolic profiles through Seahorse XFp Flux analyser and XF cell Mito Stress Test kit (Seahorse Bioscience, Billerica, MA, USA) have been reported in previous studies [26]. HCEC-B4G12 cells were incubated for 72 h after lentivirus transfection, and inoculated in eight-well assay plates with a concentration of 30,000 cells per well in complete growth medium. Before the test, HCECs were incubated in a low-buffered assay medium (Agilent Technologies) containing 5 mM of glucose and 1 mM of sodium pyruvate (pH 7.4). ATP synthase inhibitor oligomycin (1.5 µM), uncoupling agent FCCP (2 µM), and a mixture of rotenone and antimycin A (0.5 µM) were added to holes A, B, and C of the probe, respectively. Upon completion of the experiments, 20 µl of cell lysate was added to each well, and the protein concentration was detected using a bicinchoninic acid assay (Beyotime Biotechnology, Beijing, China). The oxygen consumption rate (OCR) was normalised to the protein content and presented as pmolesO2/min/µg protein.

### Statistical analysis

All data are presented as mean ± standard deviation values. GraphPad Prism version 7 software (GraphPad Software, San Diego, CA, USA) was used for the statistical analysis. Statistical comparison between the two experimental groups were analyzed using a two-tailed Student’s t-test. *P* < 0.05 was regarded statistically significant.

## Results

### Sleep deprivation contributes to corneal endothelial dysfunction in mice

**Fig. 1:**
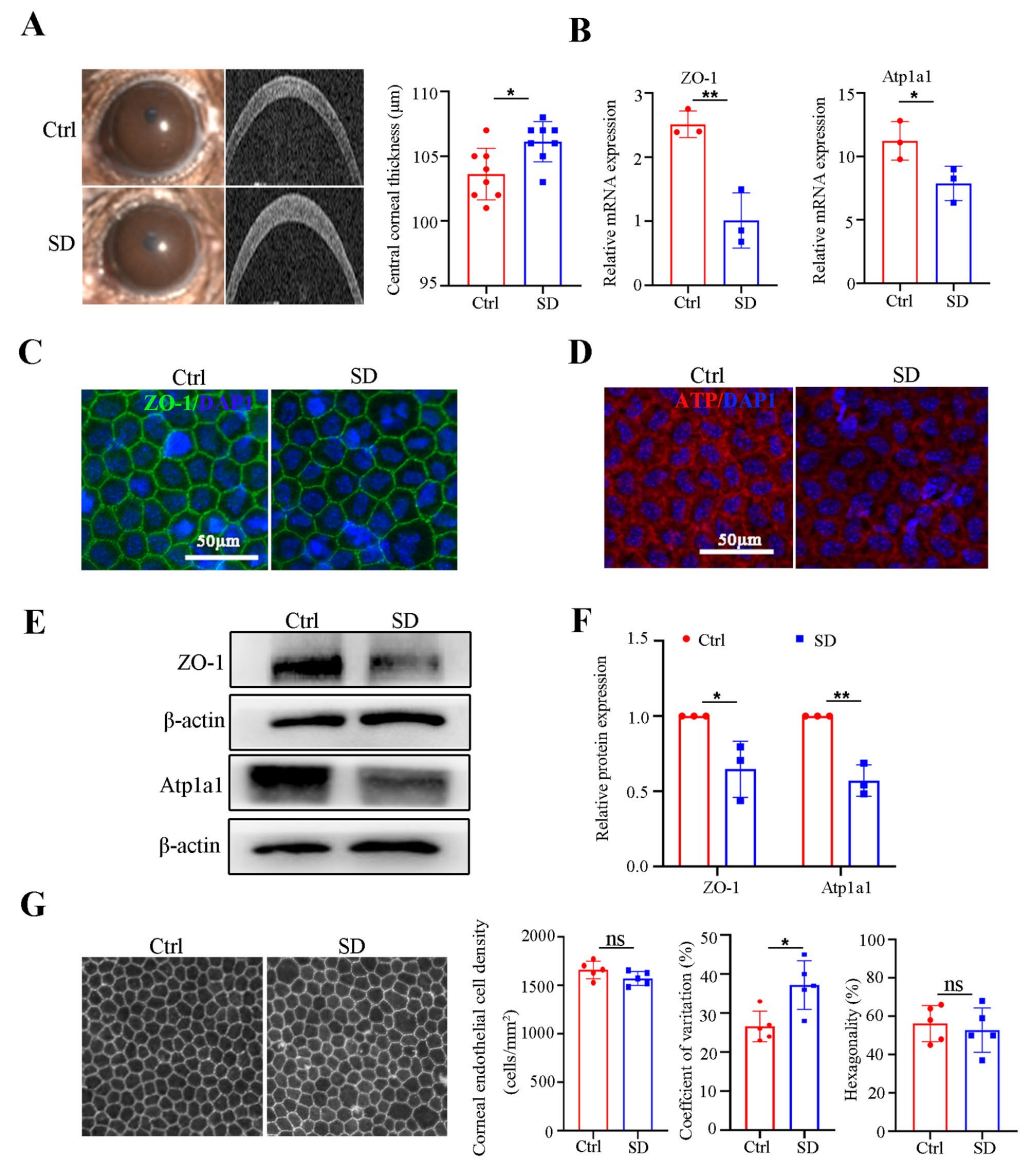
Sleep deprivation induced corneal endothelial dysfunction in mice. (**A**) Representative slit-lamp images and OCT image-based CCT analysis from control (Ctrl) and sleep deprivation (SD) mice. *n* = 8 samples per group. (**B**) Expression of functional genes (ZO-1and Atp1a1) at the mRNA levels by qRT-PCR in the corneal endothelium of the Ctrl and SD mice. Gapdh was used as an internal reference gene. *n* = 3 samples per group. (**C**, **D**) Representative confocal images of the whole mount of corneal endothelium detecting ZO-1 and Atp1a1 of the Ctrl and SD mice. *n* = 3 samples per group. (**E**, **F**) Western blot analysis of ZO-1 and Atp1a1 in the corneal endothelium from Ctrl and SD mice. b-actin was used as a loading control. *n* = 3 independent experiments. The bands of target protein and internal reference gene in each group were driven from the same gel. (**G**) Analyses of cell density, hexagonality, and coefficient of variation in the corneal endothelium of the Ctrl and SD mice. *n* = 5 samples per group. Data are presented as mean ± standard deviation. (ns, not significant, *P < 0.05, **P < 0.01)

Persistent SD has been reported to be associated with ocular diseases [27]. To investigate the effect of sleep disorders on corneal endothelial function, we first evaluated the CCT in the Ctrl and SD mice. An increased CCT is a hallmark of endothelial cell functional impairment. Compared with the Ctrl mice, CCT measurements showed increased corneal thickness in the SD mice (Fig. 1A), implying damage to corneal endothelial function. Next, we detected the mRNA expression of the tight junction marker ZO-1 and ionic pump marker Atp1a1 in the corneal endothelium. As shown in Fig. 1B, qRT-PCR results showed that the gene expressions of ZO-1 and Atp1a1 were significantly downregulated post-SD. To consolidate these findings, we applied an immunofluorescence assay and immunoblotting analysis to the corneal endothelium. Remarkably, SD led to a ~ 0.5-fold decrease in the expression of the critical functional indicators (Fig. 1C – F). Additionally, we evaluated corneal endothelial density and morphology. The SD group exhibited a 10% greater coefficient of variation than the Ctrl group, although no significant differences were observed in cell density or percentage of hexagonality between the two groups (Fig. 1G), indicating an impairment of the corneal endothelial morphology. Taken together, these results demonstrate that the SD can lead to corneal endothelial dysfunction and cellular pleomorphism.

### Sleep deprivation impairs the function of mitochondria in the mouse corneal endothelium

**Fig. 2:**
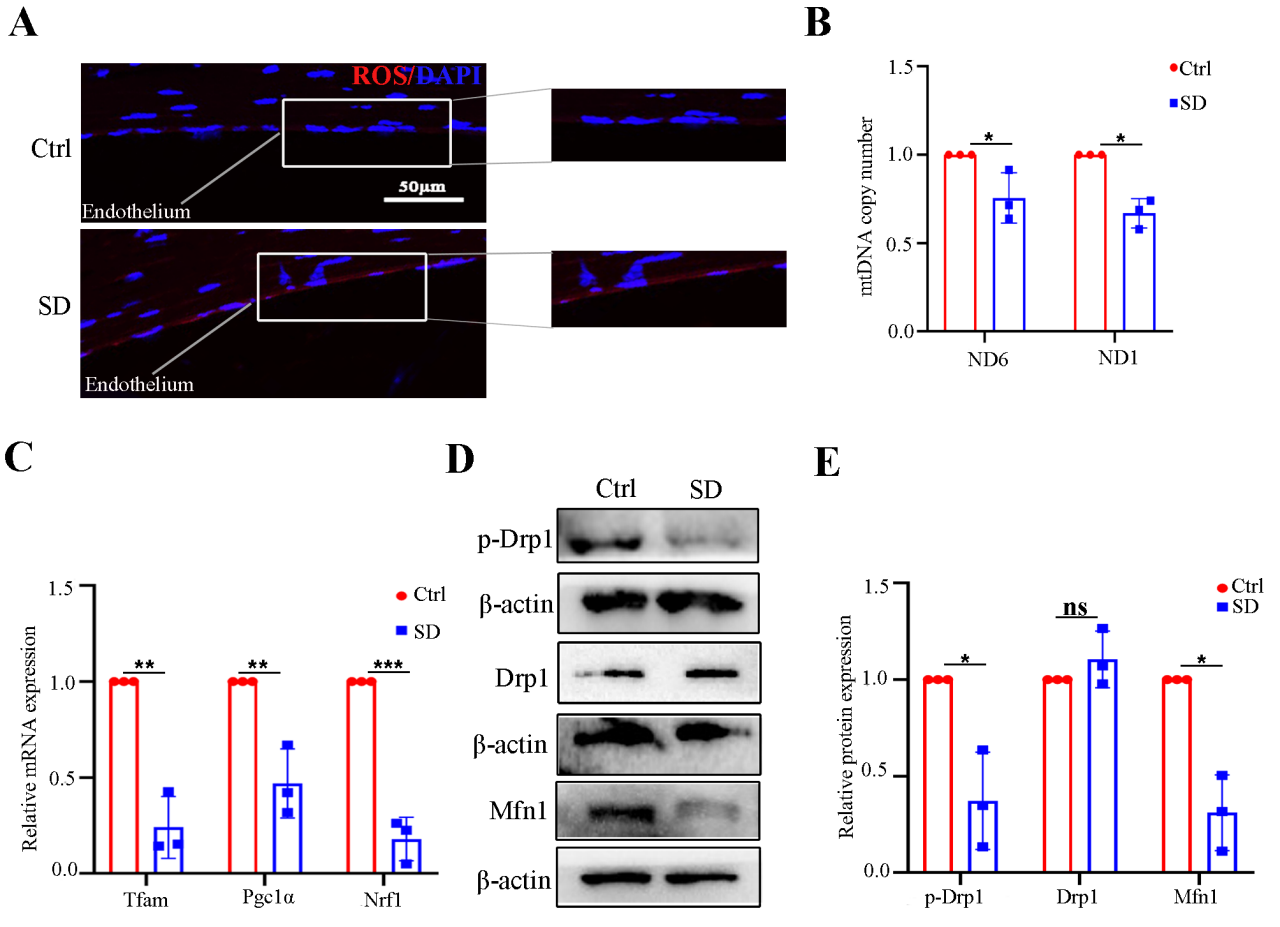
Sleep deprivation promoted mitochondrial dysfunction of the corneal endothelium in mice. (**A**) ROS staining of the corneal endothelium in the Ctrl and SD mice. *n* = 3 samples per group. (**B**) Mitochondrial abundance detected by qRT-PCR in the Ctrl and SD mice. b-globin was used as an internal reference gene. *n* = 3 samples per group. (**C**) The expressions of Tfam, Pgc1α, and Nrf1 mRNA were evaluated by qRT-PCR in the Ctrl and SD mice. Gapdh was used as an internal reference gene. *n* = 3 samples per group. (**D**, **E**) Western blot analysis of p-Drp1, Drp1, and Mfn1 in the corneal endothelium of the Ctrl and SD mice. β-actin was used as a loading control. *n* = 3 independent experiments. The bands of target protein and internal reference gene in each group were driven from the same gel. Data are presented as mean ± standard deviation. (ns, not significant, *P < 0.05, **P < 0.01, ***P < 0.001)

The corneal endothelium requires sufficient ATP supply and normal mitochondrial homeostasis to maintain corneal transparency [28]. The critical role of mitochondrial function in human corneal endothelium has been well appreciated [29]. To evaluate the effect of SD on mitochondrial function, corneal endothelia were collected from Ctrl and SD mice for analysis of oxidative stress, and mitochondrial biogenesis and dynamics. ROS accumulation is the main mechanism that leads to mitochondrial dysfunction. Indeed, we found that there was more accumulation ROS in the corneal endothelium of the SD mice (Fig. 2A). Oxidative stress plays a critical role in the accumulation of mtDNA damage. Therefore, we assessed the mtDNA copy numbers in the models. We observed that SD resulted in a remarkable decline in mtDNA copy numbers (Fig. 2B). Moreover, SD downregulated the expression level of mitochondrial biogenesis-related genes (Tfam, Pgc1a, Nrf1) (Fig. 2C), and broke the dynamic balance between mitochondrial fusion (Mfn1) and fission (Drp1) (Fig. 2D and E). Our data suggest that SD damages mouse corneal endothelial function through a mechanism that impairs mitochondrial function.

### Suppression of Bmal1 caused by sleep deprivation mediates the corneal endothelial dysfunction in mice

**Fig. 3:**
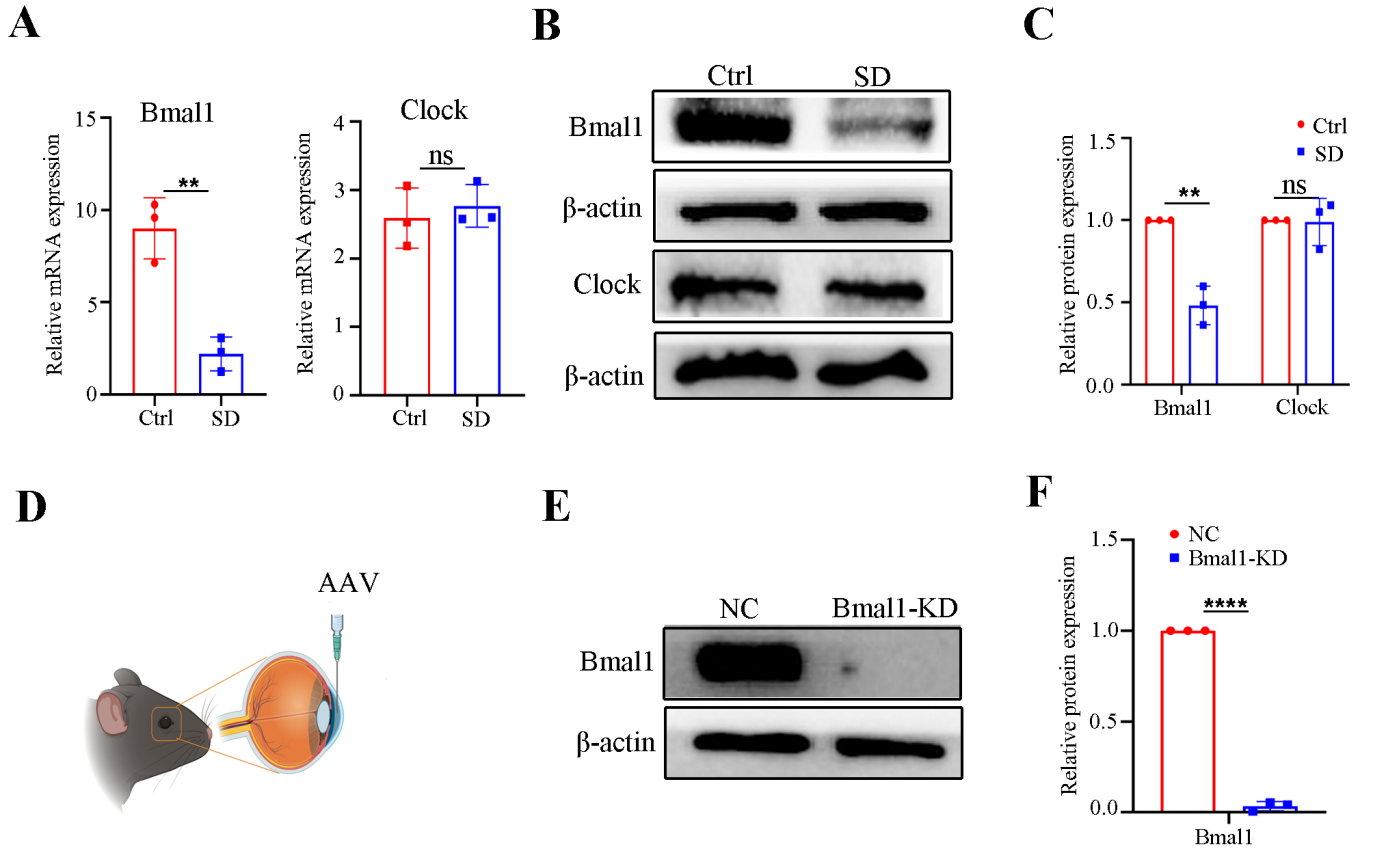
Sleep deprivation caused the downregulation of Bmal1. (**A**) Bmal1 and Clock at the mRNA levels by qRT-PCR in the corneal endothelium from the Ctrl and SD mice. Gapdh was used as an internal reference gene. *n* = 3 samples per group. (**B**, **C**) Western blot analysis of Bmal1 and Clock expressions in the corneal endothelium from the Ctrl and SD mice. β-actin was used as a loading control. *n* = 3 independent experiments. The bands of target protein and internal reference gene in each group were driven from the same gel. (**D**) Schematic diagram displaying AAV injected into the ocular anterior chambers of mice. (**E, F**) Verification of efficiency by western blot upon shRNA-mediated Bmal1-KD. *n* = 3 independent experiments. β-actin was used as a loading control. The bands of target protein and internal reference gene in each group were driven from the same gel. Data are presented as mean ± standard deviation. (ns, no significant, **P < 0.01, ****P < 0.0001)

Bmal1 and Clock, the major clock proteins, are known to be expressed in the corneal endothelium [30]. Studies have reported that sleep disorders can alter the expression of core clock proteins, leading to aggravate the progress of diseases [31]. Therefore, we assessed whether Bmal1 and Clock in the mouse corneal endothelium could be affected by SD. Surprisingly, the expression level of Bmal1 mRNA was remarkably reduced in the SD-treated corneal endothelium, while no significant difference was observed in the mRNA expression of Clock (Fig. 3A). Consistent with the mRNA expression of Bmal1, western blot results confirmed that the expression of Bmal1 at the protein level was decreased in the SD-treated corneal endothelium (Fig. 3B and C). These data imply that Bmal1 may mediate SD-associated changes in corneal endothelial function. To elucidate the role of Bmal1 in regulating corneal endothelial function, we silenced Bmal1 in the mouse corneal endothelium by directly injecting AAV-mediated shRNA that targets Bmal1 (Fig. 3D – F).

**Fig. 4:**
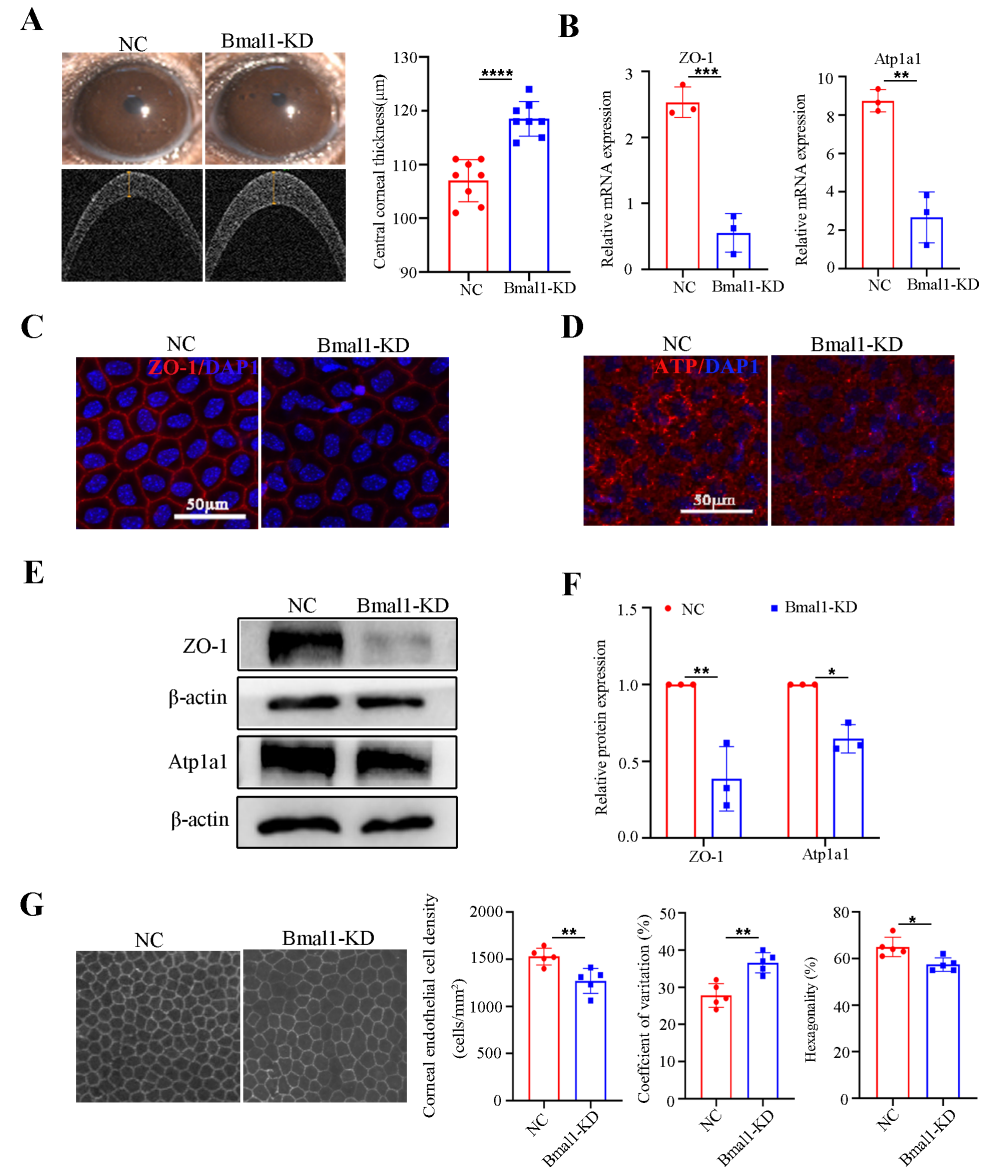
Bmal1 silencing led to impairment of the corneal endothelium. (**A**) Representative slit-lamp images and OCT image-based CCT analysis of NC and Bmal1-KD mice. *n* = 8 samples per group. (**B**) ZO-1 and Atp1a1 at the mRNA levels by qRT-PCR in the mouse corneal endothelium of the NC and Bmal1-KD groups. Gapdh was used as an internal reference gene. *n* = 3 samples per group. (**C**, **D**) Representative confocal images of the whole mount of corneal endothelium detecting ZO-1 and Atp1a1 staining from NC and Bmal1-KD mice. *n* = 3 samples per group. (**E**, **F**) Western bolt analysis for the effects of Bmal1 KD on the expressions of ZO-1 and Atp1a1 in the corneal endothelium. β-actin was used as a loading control. *n* = 3 independent experiments. The bands of target protein and internal reference gene in each group were taken from the same gel. (**G**) Analysis of cell density, hexagonality, and coefficient of variation in the corneal endothelium in NC and Bmal1-KD mice. *n* = 5 samples per group. Data are presented as mean ± standard deviation. (*P < 0.05, **P < 0.01, ***P < 0.001, ****P < 0.0001)

Compared with the NC group, CCT measurements revealed significantly increased corneal thickness in the Bmal1-KD group, implying greater damage to corneal endothelial function (Fig. 4A). To explore whether Bmal1 KD causes abnormality in corneal endothelial function, we analysed the expressions of ZO-1 and Atp1a1 by qRT-PCR, immunofluorescence, and western blot. Bmal1 silencing resulted in a decline in the mRNA expression levels of ZO-1 and Atp1a1 (Fig. 4B), accompanied by decreased fluorescence intensity, as well as the protein levels (Fig. 4C – F). Further, we found that the Bmal1 KD group presented morphological changes, including increased cell size and the loss of discernible cell borders. Specifically, the Bmal1 KD group exhibited a 26.5% lower corneal endothelial cell density, 10.5% lower hexagonality, and 10.3% higher coefficient of variation than the NC group (Fig. 4G), indicating that Bmal1 deficiency promotes impairment of the corneal endothelium in mice. Taken together, these data indicate that suppression of Bmal1 might mediate the corneal endothelial dysfunction caused by SD.

### Bmal1 overexpression ameliorates corneal endothelial dysfunction induced by SD in mice

**Fig. 5:**
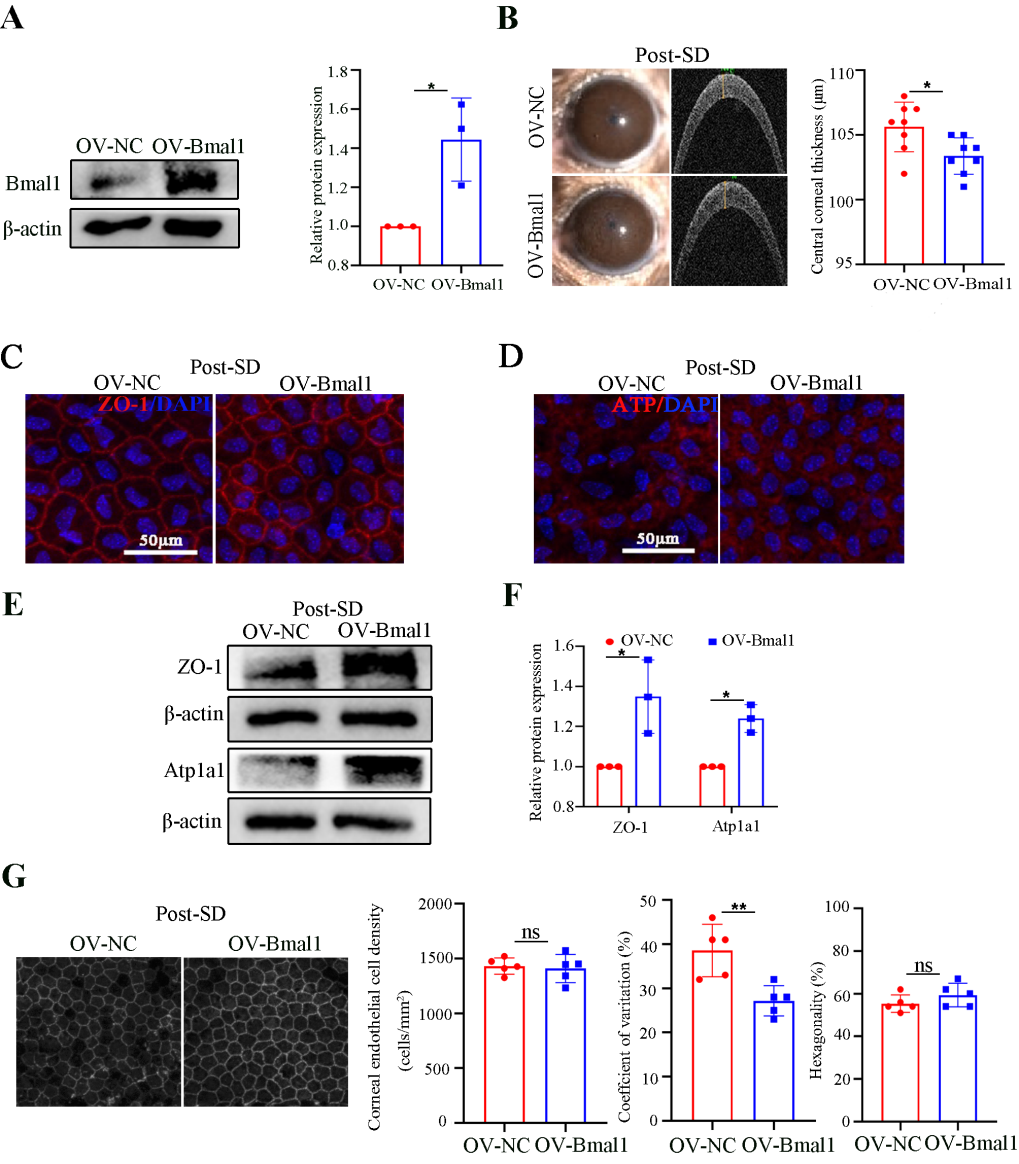
Overexpression of Bmal1 ameliorates corneal endothelial function under sleep deprivation conditions. (**A**) Verification of shRNA-mediated OV-Bmal1 efficiency by western blot analysis. β-actin was used as an internal reference gene. *n* = 3 independent experiments. The bands of target protein and internal reference gene in each group were driven from the same gel. (**B**) Representative slit-lamp images and OCT image-based CCT analysis of OV-NC and OV-Bmal1 mice post-SD. *n* = 8 samples per group. (**C**, **D**) Representative confocal images of the whole mount of corneal endothelium detecting ZO-1 and Atp1a1 in OV-NC and OV-Bmal1 mice post-SD. *n* = 3 samples per group. (**E**, **F**) Western blot analysis of ZO-1 and Atp1a1 expressions in corneal endothelium from OV-NC and OV-Bmal1 mice post-SD. β-actin was used as an internal reference gene. *n* = 3 independent experiments. The bands of target protein and internal reference gene in each group were driven from the same gel. (**G**) Analysis of cell density, hexagonality, and coefficient of variation in the corneal endothelium of OV-NC and OV-Bmal1 mice post-SD. *n* = 5 samples per group. Data are presented as mean ± standard deviation. (ns, no significant, *P < 0.05, **P < 0.01)

To determine the role of Bmal1 in protecting the corneal endothelium from SD, we overexpressed Bmal1 in the mouse corneal endothelium by directly injecting OV-Bmal1 AAV into the anterior chambers. As shown in Fig. 5A, the western blot results showed an obviously upregulated expression of Bmal1 in the OV-Bmal1 group. Next, we detected the effect of OV-Bmal1 on CCT under SD conditions. Compared with the OV-NC group, CCT measurements revealed an attenuated corneal thickness in the OV-Bmal1 group, implying greater functional preservation of the corneal endothelium (Fig. 5B). To further explore the changes in corneal endothelial function, we analysed the expressions of ZO-1 and Atp1a1 by immunofluorescence and western blot. Compared to that in OV-NC group, OV-Bmal1 retained better continuity and stronger staining of ZO-1 and Atp1a1 (Fig. 5C and D), as well as significantly upregulated expressions at the protein levels (Fig. 5E and F). We further compared the corneal endothelial density and morphology of the OV-Bmal1 and OV-NC groups. As shown in Fig. 5G, the OV-Bmal1 group exhibited a 11.4% lower coefficient of variation than in the OV-NC group. These data demonstrate that Bmal1 plays a critical role in protecting the mouse corneal endothelium from SD.

### Silencing BMAL1 contributes to mitochondrial bioenergy deficiency in HCECs

**Fig. 6:**
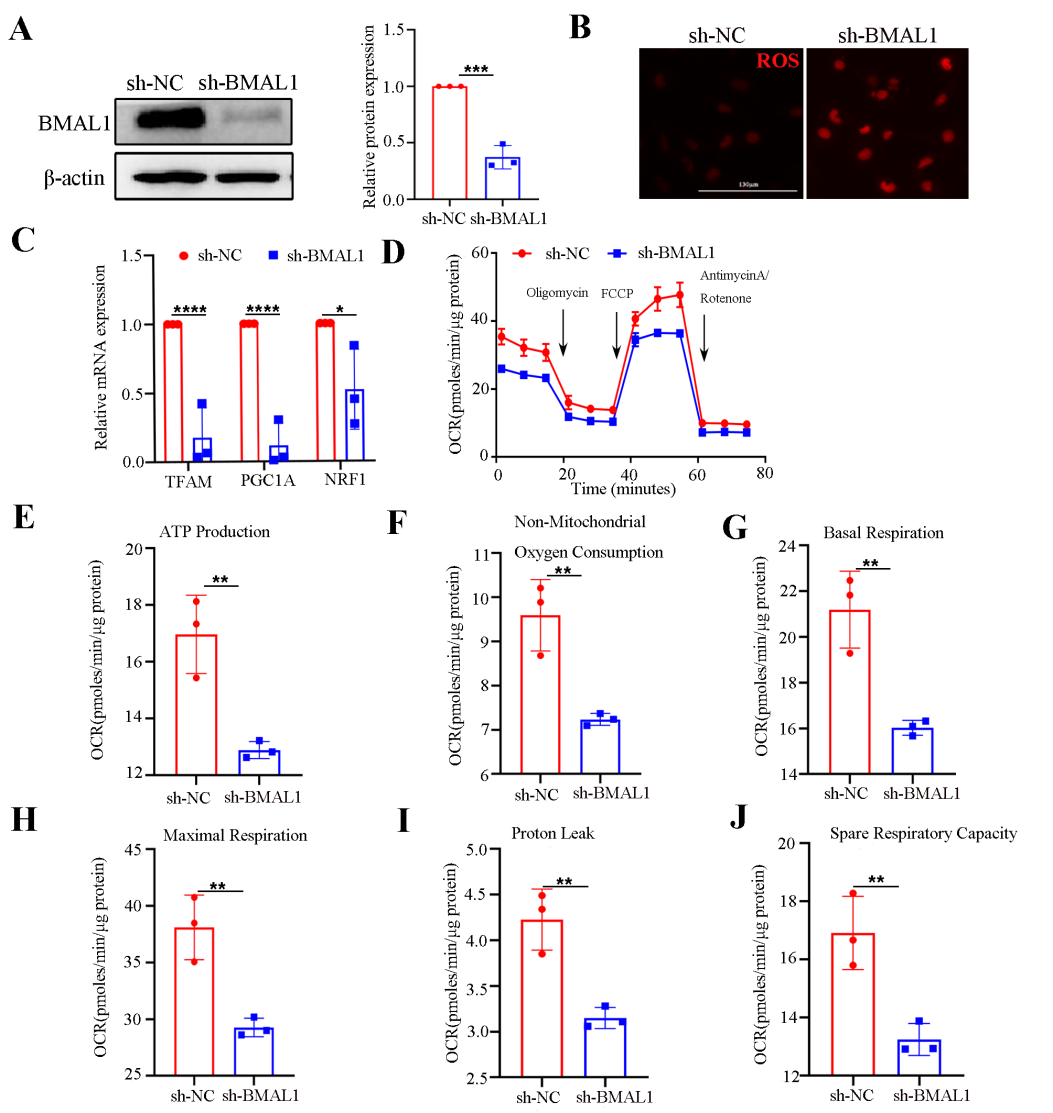
Knockdown of BMAL1 expression in HCEC-B4G12 cells induced disruption of mitochondrial bioenergetics. (**A**) Western blot analysis of BMAL1 expression in HCEC-B4G12 cells transfected with control shRNA (sh-NC) and BMAL1 shRNA (sh-BMAL1). β-actin was used as a loading control. *n* = 3 independent experiments. The bands of target protein and internal reference gene in each group were taken from the same gel. (**B**) ROS staining of HCEC-B4G12 cells in the sh-NC and sh-BMAL1 groups. *n* = 3 samples per group. (**C**) The expressions of TFAM, PGC1A, and NRF1 mRNA were examined by qRT-PCR in the sh-NC and sh-BMAL1 groups. Gapdh acted as an internal reference gene. *n* = 3 samples per group. (**D** – **J**) Mitochondrial function of HCEC-B4G12 cells treated with BMAL1 shRNA and control shRNA was assessed with the seahorse XFp analyser. After the measurement of basal OCR, ATP linked respiration and proton leak were determined following the injection of oligomycin, maximal respiration was determined after FCCP injection, reserve capacity was measured as the difference between maximal and basal respiration, and all the parameters were calculated by subtracting non-mitochondrial respiration. *n* = 3 samples per group. Data are shown as mean ± standard deviation. (*P < 0.05, **P < 0.01, ***P < 0.001, ****P < 0.0001)

Accordingly, previous data have linked dysregulation of mitochondrial function with SD. We further explored whether Bmal1 was involved in regulating mitochondrial function in the corneal endothelium. We assessed the effects of silencing BMAL1 in HCEC-B4G12 cells by lentivirus-mediated shRNA targeting knockdown BMAL1 (sh-BMAL1), with KD efficiencies validated by western blot (Fig. 6A). As expected, we observed increased ROS staining and repressed mitochondrial biogenesis-related gene transcription in the BMAL1 KD HCEC-B4G12 cells (Fig. 6B and C). We then evaluated mitochondrial bioenergetic changes using a Seahorse XFp analyzer. According to the bioenergetic profile analysis, the HCEC-B4G12 with BMAL1 KD exhibited mitochondrial impairment, including significant reduction in ATP production, maximal respiration, oxygen consumption, basal respiration, non-mitochondrial oxygen consumption, and proton leakage, as well as an approximately 25% reduction in respiratory capacity (Fig. 6D – J). These results suggest that silencing BMAL1 contributes to mitochondrial bioenergy deficiency, which may be the potential mechanism that leads to endothelial dysfunction induced by SD.

## Discussion

In the present study, we found that sleep deficiency damaged the corneal endothelial function in mice, and the underlying mechanism appeared to be related to oxidative stress and mitochondrial dysfunction. Moreover, our results showed that Bmal1 downregulation mediated corneal endothelial dysfunction induced by SD, demonstrating a new molecular mechanism of sleep loss induced damage to ocular tissues.

Insufficient sleep has broad negative effects on the health in humans [32]. Sleep disorders can alter neural synaptic plasticity by disrupting the blood-brain barrier function, leading to neuronal damage [33]. In the eye, sleep disorders have a significant risk for ocular diseases [34]. Accumulating evidence suggests that severe sleep disturbances (such as sleep apnoea) in patients lead to lower corneal endothelial density with greater pleomorphism and polymegathism [35]. However, the effects of sleep loss on corneal endothelial function have yet to be fully investigated. In the present study, our results exhibited a disruption of the tight junctional integrity of corneal endothelium in SD-treated mice, such as remarkedly decreased ZO-1 expression, which is similar to the pathological changes in corneal epithelium described in the dry eye induced by SD lasting for 10 days in mouse models [6]. Further, we found that corneal endothelial damage under SD conditions also exhibited a decrease in Na+/k + ATP pump function. Importantly, our data showed that the damage to corneal endothelial function under SD was accompanied by dysregulation of mitochondrial function. These results prompted us to further explore the underlying molecular mechanism for SD damage to corneal endothelial function.

One of most important findings of this study was that SD altered the expression of core clock gene Bmal1 in mouse corneal endothelium, which was corresponding with the observations in the extra orbital lacrimal gland after suffering from sleep deficiency [21]. Noteworthily, the downregulation expression of Bmal1 was accompanied by corneal endothelial dysfunction and mitochondrial dysfunction under SD conditions. Studies have reported that sleep loss can reduce the expression of the core clock gene Bmal1 in the pineal gland, thereby aggravating the Alzheimer’s disease neuropathology [31]. Therefore, we speculated that the alteration of Bmal1 under SD conditions could be a possible cause of dysregulation of corneal endothelial homeostasis. After knocking down Bmal1 in vivo, we observed that Bmal1 deficiency contributed to the damage of the mouse corneal endothelium, while overexpression of Bmal1 improved the corneal endothelial dysfunction caused by SD, suggesting that Bmal1 is involved in regulating corneal endothelial function under SD conditions. Taken together, our results showed that Bmal1 displayed a crucial role in maintaining corneal endothelial function under SD conditions, providing new insight into the potential link between sleep loss and damage to ocular tissues.

The mitochondrial bioenergetics are strictly regulated by the core clock genes, and Bmal1 is involved in the cellular antioxidant responses [36] and maintaining the normal mitochondrial structure and function [37, 38]. Several studies have defined the role of Bmal1 in regulating mitochondrial function in many tissues or cells. For example, Bmal1-deficient human embryonic stem cell-derived cardiomyocytes exhibit suppressed mitochondrial fission, mitophagy, and compromised cardiomyocyte function [39]. In rats, Bmal1 knockdown exacerbates the degree of mitochondrial damage and renal ischaemia-reperfusion injury [40]. In macrophages, loss- of- function of the Bmal1 gene could exacerbates mitochondrial dysfunction, energetic stress and metabolic reprogramming [41]. Our results were consistent with these previous studies showing that Bmal1 deficiency accompanies with mitochondrial dysfunction in the corneal endothelium. Upon oxidative stress, studies have reported that Bmal1-depletion increases the ROS accumulation in lens epithelial cells [16]. Consistently, our in vitro experiments showed that Bmal1 deficiency triggered oxidative stress and disrupted the mitochondrial homeostasis in the corneal endothelium, suggesting a major role of Bmal1 in mitochondrial oxidative metabolism in the corneal endothelium. Collectively, our results revealed that mitochondrial dysfunction and ROS accumulation caused by Bmal1 downregulated expression was the underlying mechanism for SD-induced endothelial damage. Understanding the role of Bmal1 in the corneal endothelium may help us to better define the pathogenesis and development of ocular diseases induced by sleep disorders. Future studies will need to explore the underlying molecular mechanism of Bmal1 involved in regulating the mitochondrial function and the antioxidant defense system in the corneal endothelium.

The study explores the impact of SD on the corneal endothelium in mice that is a crucial component in maintaining eye health, and provides valuable insights into how sleep affects ocular function, contributing to our understanding of the relationship between sleep and eye diseases. Future studies will be conducted to deeply explore the impact of SD on the corneal endothelium in humans.

There are several limitations to our study. First, it is necessary to point out that there are considerable differences between human and mouse corneal endothelium, especially in the capacity of regeneration and proliferation. Second, the laboratory mice are nocturnal animals that show the opposite pattern of behavior to human [42]. Finaly, the animal model we used is relatively single, and cumulative time of sleep deprivation may also have impact on the endothelial damage. Thus, these factors should be considered when investigating the effects of sleep deprivation on the corneal endothelium in humans.

## Conclusion

Our results showed that sleep loss had a significant negative influence on corneal endothelial homeostasis. Sleep deficiency-induced Bmal1 downregulated expression triggering ROS production and mitochondrial dysfunction was the underlying mechanism of endothelial damage, demonstrating a crucial role of Bmal1 in the corneal endothelium. These findings expand our understanding of sleep loss on ocular health and reveal an underlying mechanism for sleep disorders leading to damage of corneal endothelium.

### Electronic supplementary material

Below is the link to the electronic supplementary material.

Supplementary Material 1

Supplementary Material 2

## Data Availability

All data generated or analyzed during this study are included in this published article.

## Abbreviations

SD: Sleep deprivation
CCT: Central corneal thickness
ROS: Reactive oxygen species
OCT: Optical coherence tomography
HCEC-B4G12: Human corneal endothelial cell line B4G12
KD: Knockdown
NC: Negative control
OV: Overexpression
Ctrl: Control
OCR: Oxygen consumption rate
