# Correction: Sleep deprivation induces corneal endothelial dysfunction by downregulating Bmal1

**DOI:** 10.1186/s12886-024-03545-z

**Published:** 2024-07-01

**Authors:** Yani Wang, Qun Wang, Shengqian Dou, Qingjun Zhou, Lixin Xie

**Affiliations:** 1 Eye Institute of Shandong First Medical University, Qingdao Eye Hospital of Shandong First Medical University, 5 Yan er dao Road, Qingdao, 266071 China; 2 State Key Laboratory Cultivation Base, Shandong Provincial Key Laboratory of Ophthalmology, Shandong First Medical University, Shandong, China; 3 School of ophthalmology, Shandong First Medical University, Shandong, China

**Correction: BMC Ophthalmol 24, 268 (2024)**

10.1186/s12886-024-03524-4

In this article [1], Yani Wang and Qun Wang should have been denoted as equally contributing authors.

The original article has been corrected.

## References

[1] Wang Y, Wang Q, Dou S (2024). Sleep deprivation induces corneal endothelial dysfunction by downregulating Bmal1. BMC Ophthalmol.

