# Supplementary material for: Sleep deprivation induces corneal endothelial dysfunction by downregulating Bmal1
Source: BMC Ophthalmol. 2024 Jun 21;24:268. doi: 10.1186/s12886-024-03524-4 (PMC11191275; doi:10.1186/s12886-024-03524-4)
Supplement: Supplementary file 1 — Supplementary Material 1 [file 12886_2024_3524_MOESM1_ESM.pdf]

Supplementary Table S1. Primer sequences for the targeted genes in qRT-PCR

Gene	Forward primer (5'--3')	Reverse primer (3'--5')
m-ZO-1	CCAGGGTGAAGGCAACTCA	GGGAAACTTTTCTGCGGATAGA
m-Atp1a1	TAGCGGGAGATGCCTCTGAGT	TTGGGTTCTTGTGAATGGACAA
m-Bmal1	CAACCTTCCCGCAGCTAACA	CCGCGATCATTCGACCTATT
m-Clock	ACACAGCCAGCGATGTCTCA	CTGCCGATGAATATTTGCCTCTA
m-mtND1	CCCTACCAATACCACACCCATT	GGGCTACGGCTCGTAAAGCT
m-mtND6	CATACATCAACCAATCTCCCAAAC	GACTGCTATAGCTACTGAGGAATATCCA
m- β-globin	GGACCCAGCGGTACTTTGATAG	TGGCAAAGGTGCCCTTGA
m-Tfam	CGCATCCCCTCGTCTATCAG	TGAAAGTTTTGCATCTGGGTGTT
m-Pgc1α	GAAGAGCGCCGTGTGATTTAC	ACGGTAGGTGATAAACCATAGC
m-Nrf1	GGAAACGGCCTCATGTGTTT	TGGGTTTGGAGGGTGAGATG
m-Gapdh	GCCACCCAGAAGACTGTGGAT	GGAAGGCCATGCCAGTGA
h-GAPDH	CATGTTCATCATGGGTGTGAA	GGCATGGACTGTGGTCATGAT
h-PGC1A	TTTTCTCGACACAGGTCGTGTT	TCTCACATACAAGGGAGAATTTCG
h-NRF1	TCAAGTACTCTACAGGTCGGGGA	TTCCCGCCCATGCTGTTTA
h-TFAM	TTTACCGAGGTGGTTTTCATCTG	CGCTGGGCAATTCTTCTAATTAG

Supplementary Table S2. Antibodies for western blot and immunofluorescence

Primary antibody	Dilution concentration	Supplier	Code
Anti-Bmal1	WB (1/1000)	Immunoway	YT5423
Anti-Clock	WB (1/1000)	Cell Signaling	D45B10
Anti-β-actin	WB (1/1000)	Proteintech	20536-1-AP
Anti-ZO-1	WB(1:1000) IF (1:200)	Invitrogen	40-2200
Anti-Atp1a1	WB (1/5000)	Proteintech	14418-1-AP
Anti-phoso-Drp1	WB (1/1000)	Affinity	AF8470
Anti-Drp1	WB (1/1000)	Affinity	DF7037
Anti-Mfn1	WB (1/500)	Proteintech	13798-1-AP
Anti- Atp1a1	IF (1/200)	Abcam	ab76020
Goat-anti-rabbit IgG	WB (1/2000)	Proteintech	SA00001-2
Alexa Fluor 488 goat anti-rabbit IgG H&L	IF (1/200)	Invitrogen	35552
ALexa Fluor 647 goat anti-rabbit IgGH&L	IF (1/200)	Abcam	ab150083

Abbreviations: WB, Western blot; IF, immunofluorescence
