# Supplementary figures and images for: Sleep deprivation induces corneal endothelial dysfunction by downregulating Bmal1
Source: BMC Ophthalmol. 2024 Jun 21;24:268. doi: 10.1186/s12886-024-03524-4 (PMC11191275; doi:10.1186/s12886-024-03524-4)

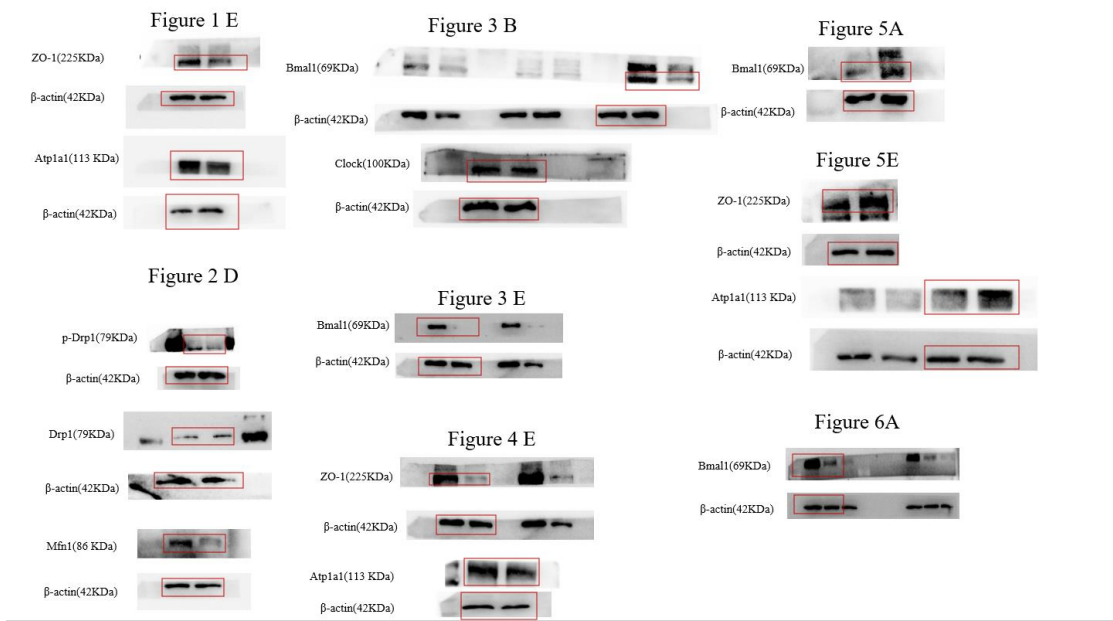

Supplement: Supplementary file 2 — Supplementary Material 2 [file 12886_2024_3524_MOESM2_ESM.pdf]
